# Albumin and C-reactive protein relate to functional and body composition parameters in patients admitted to geriatric rehabilitation after acute hospitalization: findings from the RESORT cohort

**DOI:** 10.1007/s41999-022-00625-5

**Published:** 2022-03-02

**Authors:** Jeanine M. Van Ancum, Camilla S. L. Tuttle, René Koopman, Mirjam Pijnappels, Carel G. M. Meskers, Sanjoy K. Paul, Wen Kwang Lim, Esmee M. Reijnierse, Gordon S. Lynch, Andrea B. Maier

**Affiliations:** 1 Department of Human Movement Sciences, @AgeAmsterdam, Faculty of Behavioural and Movement Sciences, Amsterdam Movement Sciences, Vrije Universiteit Amsterdam, Amsterdam, The Netherlands; 2 Department of Medicine and Aged Care, @AgeMelbourne, The Royal Melbourne Hospital, The University of Melbourne, Centre for Medical Research Building, 300 Grattan Street, Parkville, VIC 3010 Australia; 3 Centre for Muscle Research, Department of Physiology, School of Biomedical Sciences, The University of Melbourne, Melbourne, VIC Australia; 4 Department of Rehabilitation Medicine, Amsterdam Movement Sciences, Amsterdam UMC, Vrije Universiteit Amsterdam, de Boelelaan 1117, Amsterdam, The Netherlands; 5 Melbourne EpiCentre, University of Melbourne and Melbourne Health, Melbourne, VIC Australia; 6 Healthy Longevity Translational Research Program, Yong Loo Lin School of Medicine, National University of Singapore, Singapore, Singapore; 7 Centre for Healthy Longevity @AgeSingapore, National University Health System, Singapore, Singapore

**Keywords:** Inflammation, Activities of daily living, Physical functional performance, Muscle strength, Sarcopenia

## Abstract

**Purpose:**

Albumin and C-reactive protein (CRP) are non-specific markers of inflammation, which could affect muscle tissue during acute hospitalization. We investigated the association between albumin and CRP during acute hospitalization with functional and body composition parameters in patients admitted to geriatric rehabilitation.

**Methods:**

The REStORing Health of Acutely Unwell AdulTs (RESORT) cohort includes geriatric rehabilitation patients assessed for change in activities of daily living (ADL, using the Katz index) during acute hospitalization, and subsequently for Katz ADL, gait speed (GS), handgrip strength (HGS) and skeletal muscle mass index (SMI) at geriatric rehabilitation admission. Albumin and CRP average (median), variation (interquartile range), and maximum or minimum were collected from serum samples, and were examined for their association with functional and body composition parameters using multivariable linear regression analysis adjusted for age, sex and length of acute hospital stay.

**Results:**

1769 Inpatients were included for analyses (mean age 82.6 years ± 8.1, 56% female). Median length of acute hospitalization was 7 [IQR 4, 13] days and median number of albumin and CRP measurements was 5 [IQR 3, 12] times. ADL declined in 89% of patients (median − 3 points, IQR − 4, − 2). Lower average albumin, higher albumin variation and lower minimum albumin were associated with larger declines in ADL and with lower ADL, GS, HGS and SMI at geriatric rehabilitation admission. Higher average and maximum CRP were associated with lower GS.

**Conclusion:**

Inflammation, especially lower albumin concentrations, during acute hospitalization is associated with lower physical function at geriatric rehabilitation admission.

**Supplementary Information:**

The online version contains supplementary material available at 10.1007/s41999-022-00625-5.

## Key summary points

**Aim:**

To investigate the association between albumin and C-reactive protein during acute hospitalization with functional and body composition parameters in patients admitted to geriatric rehabilitation.

**Findings:**

Lower average albumin, higher albumin variation and lower minimum albumin were associated with larger declines in physical function during acute hospitalization and with lower functional and body composition parameters at geriatric rehabilitation admission. C-reactive protein, was only partly associated with lower gait speed at geriatric rehabilitation admission.

**Message:**

Inflammation during acute hospitalization, especially lower albumin concentrations, relates to declined physical function and low functional and body composition parameters upon geriatric rehabilitation admission.

**Supplementary Information:**

The online version contains supplementary material available at 10.1007/s41999-022-00625-5.

## Introduction

Acute hospitalization influences muscle homeostasis and physical function negatively [1–3]. Over one-third of older patients experience loss in activities of daily living (ADL) during hospitalization [2, 4, 5]. Inflammation is likely an important contributor to muscle wasting following acute hospitalization, and a possible target in the prevention of disability [6]. Acute systemic inflammation, characterized by low albumin and high C-reactive protein (CRP) serum levels, is often observed in acutely ill hospitalized patients and can contribute to high levels of muscle wasting [7]. This inflammation can lead to a catabolic state [8], which in turn can cause muscle degeneration through myonuclear apoptosis, alterations in muscle protein turnover and impaired satellite cell function [7, 9, 10].

Albumin and CRP are proteins synthesized in the liver and respond to pro-inflammatory cytokines [11–14]. Albumin has been suggested as a marker for the nutritional state, but this has been questioned [15]. More recent, albumin has been used as a marker for overall severity of disease; however, the ability to maintain normal levels of albumin may also indicate a level of protection against physiological stress caused by inflammation [12]. Albumin could, therefore, be used as a marker of inflammation: albumin regulates plasma oncotic pressure, and is an antioxidant that moderates the inflammatory response by binding pro-inflammatory molecules [16, 17]. In sepsis and acute illness, the albumin transcapillary escape rate is increased, and the synthesis rate in the liver is increased to a lesser extent, resulting in an altered distribution over fluid compartments and decreased serum albumin [17]. As such, low levels of albumin may increase the risk of catabolism in an individual. Lower albumin concentrations have shown a strong relation with sarcopenia in older adults [18, 19], and have shown to synergistically contribute to increased disability [20]. Lower levels of albumin and higher levels of CRP are associated with a decline in ADL, gait speed (GS), handgrip strength (HGS) and skeletal muscle mass index (SMI) after 5–10 years of follow-up in community-dwelling older adults [21–26].

There is limited knowledge about how inflammation experienced during acute hospitalization influences functional and body composition parameters in patients admitted to geriatric rehabilitation. We investigated the associations of albumin and CRP during acute hospitalization with functional and body composition parameters (ADL, GS, HGS, SMI) in patients admitted to geriatric rehabilitation.

## Methods

### Study design

REStORing health of acutely unwell adulTs (RESORT) is an observational, prospective and longitudinal inception cohort study that commenced recruitment in October 2017 at the Royal Melbourne Hospital (Melbourne, Victoria, Australia). A description of the protocol is accessible elsewhere [27]. The cohort included 1890 patients admitted to geriatric rehabilitation wards from October 16th 2017 and discharged by March 18th 2020. The study was approved by the Melbourne Health Human Research Ethics Committee (no. HREC/17/MH/103). Written informed consent was obtained from the patients or a nominated proxy. Patients were excluded (*n* = 446, 16.6%) if they were transferred to another healthcare service before consent was obtained, were unable to give informed consent (e.g., severe dementia, delirium) and did not have a nominated proxy, or were receiving palliative care. A total of 356 patients (13.2%) refused to participate in the study. Patients were assessed within 48 h of admission to geriatric rehabilitation and within 48 h of discharge. A Comprehensive Geriatric Assessment (CGA) [28] was performed in every patient as part of usual care by trained medical, nursing and allied health professionals and included physical, cognitive, functional and social domains.

### Data collection

#### Patient characteristics

Demographic data were collected through a self-reported survey at admission to geriatric rehabilitation and included age, sex, living status, use of a walking aid, cognitive impairment and experiencing a fall in the preceding year. Multimorbidity was assessed using the Charlson Comorbidity Index (CCI) [29], risk of malnutrition using the Malnutrition Screening Tool (MST) [30], Global Leadership Initiative on Malnutrition (GLIM) malnutrition prevalence [31], and frailty status using the Rockwood Clinical Frailty Scale (CFS) [32]. Height was measured upright if patients were able to stand, otherwise height was estimated from the knee height using the Longitudinal Aging Study Amsterdam (LASA) formula (male = 74.48 + [2.03 × knee height] − [0.15 × age], female = 68.74 + [2.07 × knee height] − [0.16 × age]) [33]. Weight was measured using a weighing scale or weighing chair. Body mass index was calculated from height and weight. Acute admission diagnosis, number of medication, length of stay (LOS) for acute hospitalization and geriatric rehabilitation were collected from medical records.

#### Functional and body composition parameters

ADL was scored by trained occupational therapists using the self-reported Katz index [34] 2 weeks before acute hospitalization and at admission to geriatric rehabilitation. The score on the Katz ADL index ranges from 0 to 6 points, with 0 points indicating full dependency and 6 points indication full independency. Change in ADL was calculated subtracting the score at geriatric rehabilitation admission with the score 2 weeks before acute hospitalization.

GS, HGS and SMI were measured at geriatric rehabilitation admission: GS (m/s) was measured using the 4-m walk test at usual pace from a standing start, following the protocol of the Short Physical Performance Battery [35]. The fastest trial out of two attempts was used for analysis. HGS (kg) was measured using a hydraulic handheld dynamometer (Jamar, Sammons Preston, Inc. Bolingbrook, IL, USA) sitting upright in a chair, with elbows unsupported at an angle of 90° [36]. Where patients were confined to bed, HGS was measured in supine position with elbows unsupported in an angle of 30°. Repeated measurements were performed in the same position with three attempts per hand, alternating between hands [37]. The maximum score out of six attempts was used for analysis. SMI (kg/m^2^) was measured using direct segmental multi-frequency bioelectrical impedance analysis (DSM-BIA, InBody S10, Biospace Co., Ltd, Seoul) [38].Contraindications for DSM-BIA measurement included pacemaker or any electronic internal medical device, plasters or bandages that could not be removed from the positioning place of the electrodes, amputated arm and/or leg or contact isolation.

#### Inflammatory markers

Albumin and CRP obtained during routine clinical care were used in the present analysis and retrieved from medical records. Albumin (g/L) was measured in serum samples by Albumin BCP assay on the Architect *c*Systems. High-sensitivity CRP (mg/L) was measured in serum samples by Multigent CRP Vario assay for quantitative immunoturbidimetric determination using the Architect *c*Systems.

Albumin was expressed as: (1) the median value (average albumin); (2) the interquartile range (albumin variation); and (3) the minimum albumin calculated over all measurements taken during acute hospitalization. CRP was expressed as: (1) the median value (average CRP); (2) the interquartile range (CRP variation); and (3) the maximum CRP. Clusters were formed combining high versus low levels of minimum albumin and albumin variation with high versus low levels of average CRP. Median values were used to categorize high versus low, as predefined cutoff values for high versus low average, variation and minimum are not available.

### Statistical analysis

**Fig. 1:**
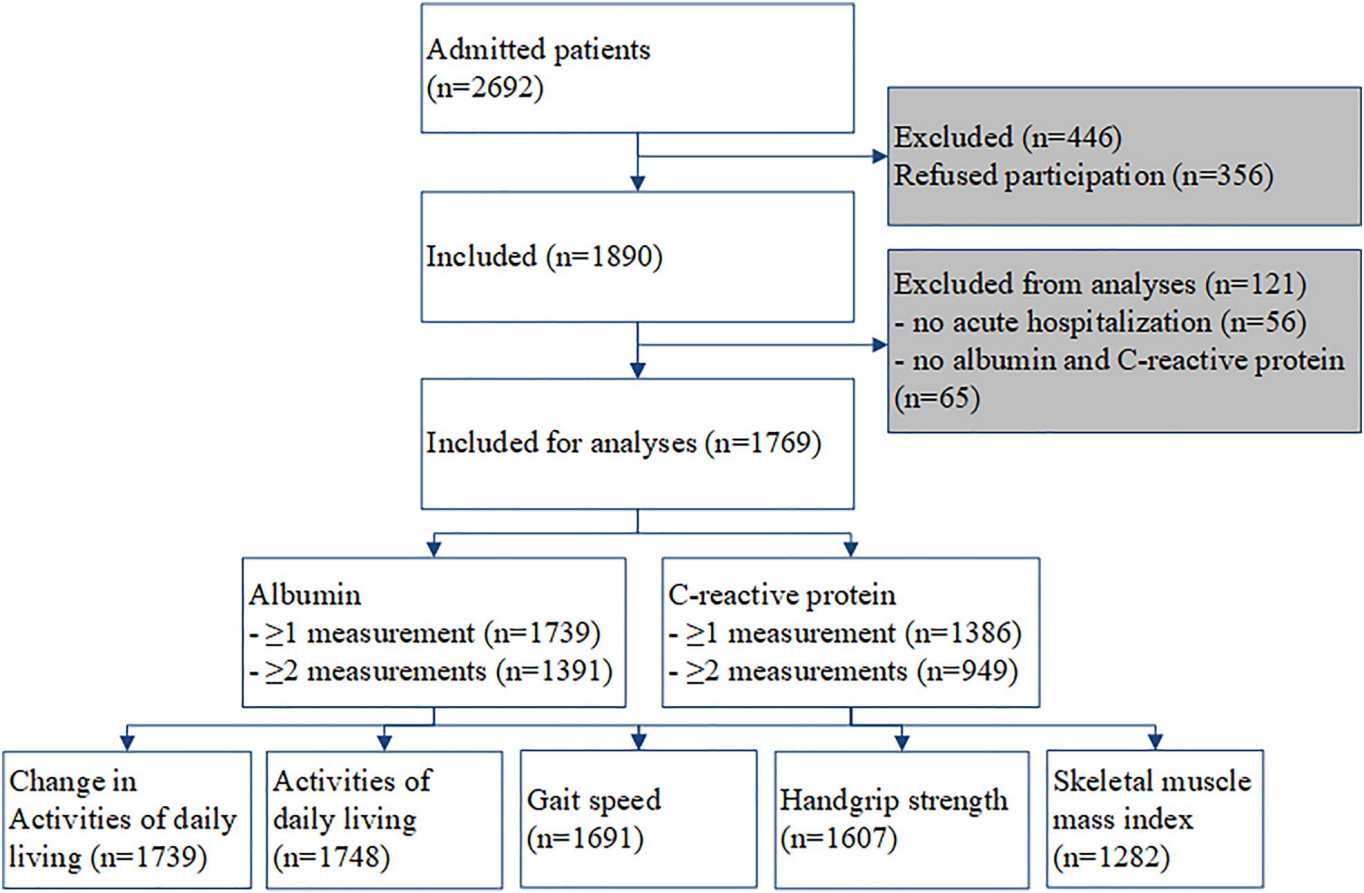
Flowchart of data availability of RESORT

**Fig. 2:**
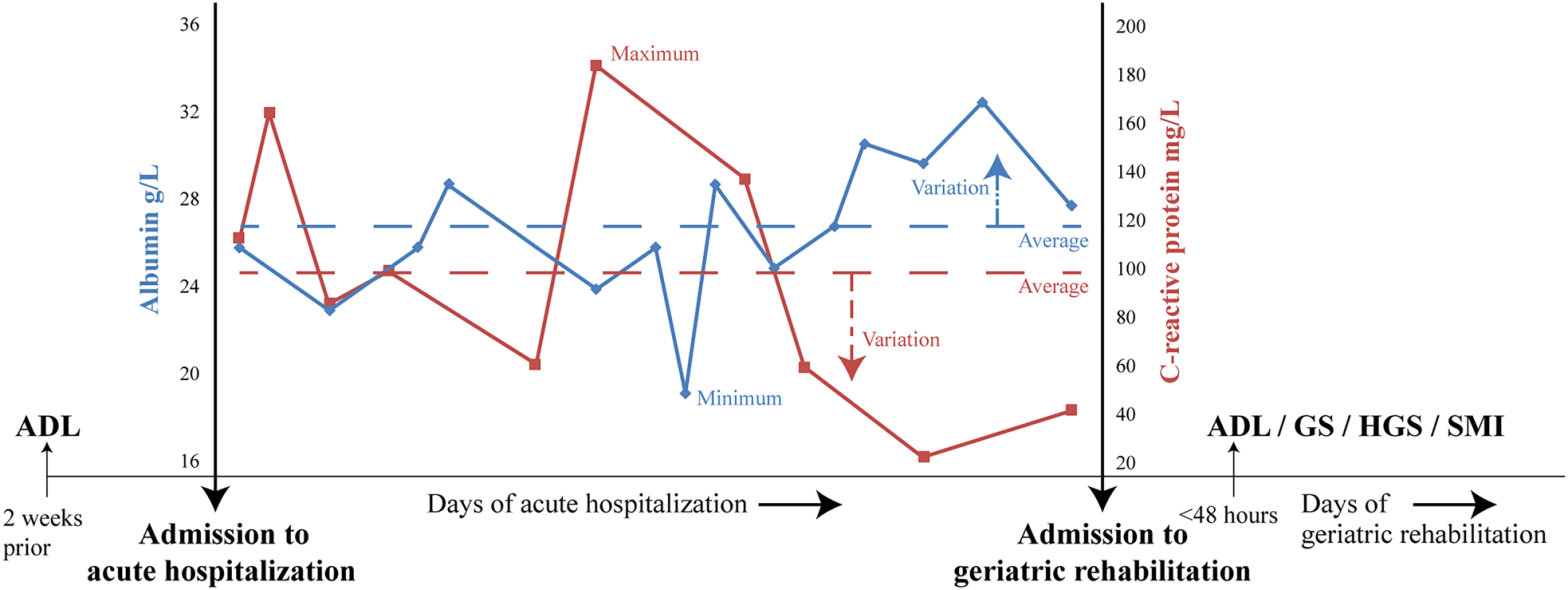
Overview of albumin, C-reactive protein and functional and body composition parameters during acute hospitalization and at geriatric rehabilitation admission. *ADL* activities of daily living, *GS* gait speed, *HGS* handgrip strength, *SMI* skeletal muscle mass index. Dashed lines indicate the average of albumin and C-reactive protein concentrations. Dashed arrows indicate the variation of albumin and C-reactive protein concentrations

The analysis included 1769 out of 1890 patients (Fig. 1). Figure 2 visualizes the timeline of data collection. Data was presented as number (%), mean (SD) or median [IQR] as appropriate and unadjusted residuals of all models were checked for normality. Potential selection bias of patients with zero, one, or ≥ two measurements of albumin and CRP was investigated using one-way ANOVA (normal distribution), Mann–Whitney *U* test (skewed distribution) or Chi-square test (categorical variables).

A multivariable linear regression analysis was used to determine the association between the average, variation and minimum albumin and maximum CRP levels with change in ADL, ADL, GS, HGS and SMI. These models were adjusted for age, sex and length of acute hospital stay. The model with change in ADL was additionally adjusted for baseline ADL 2 weeks before acute hospitalization.

Minimum albumin and average CRP were combined into clusters. Clusters consisted of low versus high minimum albumin and high versus low average CRP, with the most favorable cluster including high minimum albumin and low average CRP, and the least favorable cluster including low minimum albumin and high average CRP based on previous literature [21–26]. Clusters consisting of high versus low albumin variation and high versus low average CRP were included as Online Resource 1. The associations between the clusters with change in ADL, ADL, GS, HGS and SMI were analyzed using multivariable linear regression models with dummy variables of the clusters, using the same adjustments as described above. Results of the clusters were visualized as bar charts with unstandardized predicted medians using GraphPad Prism for Windows (version 8.0. GraphPad Software Inc.). Associations were considered statistically significant if *p* < 0.05. The Statistical Package for the Social Sciences was used for all analyses (IBM SPSS Statistics for Windows, Version 23.0. Armonk, NY, IBM Corp).

## Results

Characteristics of the RESORT patients are shown in Table 1. Mean age was 82.6 years (SD 8.1), 984 patients (55.6%) were female, 71.8% used a walking aid and 65.8% had experienced a fall in the preceding year. From 2 weeks before acute hospitalization to geriatric rehabilitation admission, 89% of patients experienced a decline in ADL with a median of − 3 points [IQR − 4, − 2]. Online Resource 2 shows the characteristics of patients stratified by zero, one or ≥ two number of albumin and CRP measurements (albumin median 4 [IQR 2, 8] number of measurements during acute hospitalization, CRP median 2 [IQR 1, 5] number of measurements). Compared to patients with zero or one measurement of albumin or CRP, patients with ≥ two measurements were more likely to be male, malnourished, more frail, use more medication, have multimorbidity, a longer acute and geriatric rehabilitation LOS, a lower average and minimum albumin, a higher average and maximum CRP, and a lower ADL and HGS.

**Table 1:** RESORT patient characteristics

	*n*	Total (*n* = 1769)
Age, years	1769	82.6 (8.1)
Sex, female, *n* (%)	1769	984 (55.6)
Acute admission diagnosis, *n* (%)	1769	
Infection		115 (6.5)
Neurological		263 (14.9)
Gastrointestinal		99 (5.6)
Respiratory		124 (7.0)
Cardiac		137 (7.7)
Musculoskeletal		824 (46.6)
Other		207 (11.7)
BMI, kg/m^2^	1722	26.8 (6.3)
Living independently, *n* (%)	1769	1653 (93.4)
CCI score	1769	2.79 (2.32)
Cognitive impairment, *n* (%)	1769	1160 (65.6)
MST score	1746	1 [0–2]
GLIM malnourished, *n* (%)	1427	805 (56.4)
CFS score	1607	6 [5–7]
Use of walking aid, *n* (%)	1756	1261 (71.8)
Fall in previous 12 months, *n* (%)	1738	1144 (65.8)
Medication, number	1769	9.6 (4.3)
LOS acute hospitalization, days	1769	7 [4–13]
LOS geriatric rehabilitation, days	1769	20 [13–32]
Albumin, g/L		
Average	1739	31 [27–34]
Variation	1391	2 [1–4]
Minimum	1739	29 [24–33]
CRP, mg/L		
Average	1386	32.4 [9.3–80.7]
Variation	949	24.0 [6.2–59.1]
Minimum	1386	50.2 [11.8–149.0]
ADL 2 weeks before acute hospitalization	1741	6 [4–6]
ADL at geriatric rehabilitation admission	1748	2 [1, 2]

	*n*	Total (*n* = 1769)	
		Male	Female
GS, m/s	1691	0.27 (0.31)	0.21 (0.26)
HGS, kg	1607	19.2 (9.7)	12.2 (6.9)
SMI, kg/m^2^	1282	9.45 (1.44)	8.50 (1.33)

All variables are presented as mean (SD) or median [IQR], unless indicated otherwise

*ADL* activities of daily living, *BMI* Body mass index, *CCI* Charlson comorbidity index, *CFS* Clinical frailty scale, *CRP* C-reactive protein, *GLIM* Global leadership initiative on malnutrition, *GS* gait speed, *HGS* handgrip strength, *IQR* interquartile range, *LOS* length of stay, *MST* malnutrition screening tool, *SD* standard deviation, *SMI* Skeletal muscle mass index

Table 2 shows the associations between albumin and CRP with functional and body composition parameters. Lower average albumin was associated with lower change in ADL, ADL, GS, HGS and SMI. For every one g/L lower average albumin, patients had 0.034 points larger decline in ΔADL, 0.030 points lower ADL, 0.009 m/s lower GS, 0.119 kg lower HGS, and 0.018 kg/m^2^ lower SMI. Higher albumin variation was associated with lower change in ADL, ADL and GS. For every one g/L higher variation albumin, patients had 0.095 points larger decline in ΔADL, 0.064 points lower ADL, and 0.012 m/s lower GS. Lower minimum albumin was associated with lower change in ADL, ADL, GS, HGS and SMI. For every one g/L lower minimum albumin, patients had 0.034 points larger decline in ΔADL, 0.028 points lower ADL, 0.007 m/s lower GS, 0.101 kg lower HGS and 0.022 kg/m^2^ lower SMI. Higher average and maximum CRP were associated with lower GS. For every one mg/L higher average and maximum CRP, patients had 0.001 and 0.000 m/s lower GS, respectively. No statistically significant associations were observed for CRP and change in ADL, ADL, HGS and SMI.

**Table 2:** Associations between albumin and CRP during acute hospitalization with change in ADL, ADL, GS, HGS and SMI at geriatric rehabilitation admission

	*n*	Change in ADL* B (95% CI)	*n*	ADL B (95% CI)	*n*	GS B (95% CI)	*n*	HGS B (95% CI)	*n*	SMI B (95% CI)
Albumin g/L										
Average	1710	**0.034 (0.019, 0.048)**	1719	**0.030 (0.014, 0.045)**	1661	**0.009 (0.006, 0.012)**	1580	**0.119 (0.032, 0.206)**	1259	**0.018 (0.001, 0.034)**
Variation	1367	− **0.095 (**− **0.137, **− **0.052)**	1376	− **0.064 (**− **0.109, **− **0.019)**	1328	− **0.012 (**− **0.021, **− **0.003)**	1255	− 0.151 (− 0.415, 0.113)	997	− 0.021 (− 0.071, 0.029)
Minimum	1710	**0.034 (0.021, 0.047)**	1719	**0.028 (0.014, 0.042)**	1661	**0.007 (0.005, 0.010)**	1580	**0.101 (0.023, 0.179)**	1259	**0.022 (0.008, 0.037)**
CRP mg/L										
Average	1357	− 0.002 (− 0.003, 0.000)	1366	− 0.001 (− 0.002, 0.000)	1325	− **0.001 (**− **0.001, **− **0.000)**	1249	− 0.002 (− 0.010, 0.005)	995	0.000 (− 0.002, 0.001)
Variation	930	− 0.001 (− 0.003, 0.001)	936	0.000 (− 0.002, 0.002)	906	− 0.000 (− 0.001, 0.000)	849	0.003 (− 0.009, 0.015)	660	0.000 (− 0.002, 0.002)
Minimum	1357	− 0.001 (− 0.002, 0.000)	1366	− 0.001 (− 0.001, 0.000)	1325	− **0.000 (**− **0.000, **− **0.000)**	1249	− 0.001 (− 0.006, 0.004)	995	0.000 (− 0.001, 0.001)

All models are adjusted for age, sex and length of acute hospital stay. Bold indicates statistical significant results (*p* ≤ 0.05)

*ADL* activities of daily living, *B* beta, *CI* confidence interval, *CRP* C-reactive protein, *GS* gait speed, *HGS* handgrip strength, *SMI* Skeletal muscle mass index

*Additionally adjusted for baseline ADL 2 weeks before acute hospitalization

**Fig. 3:**
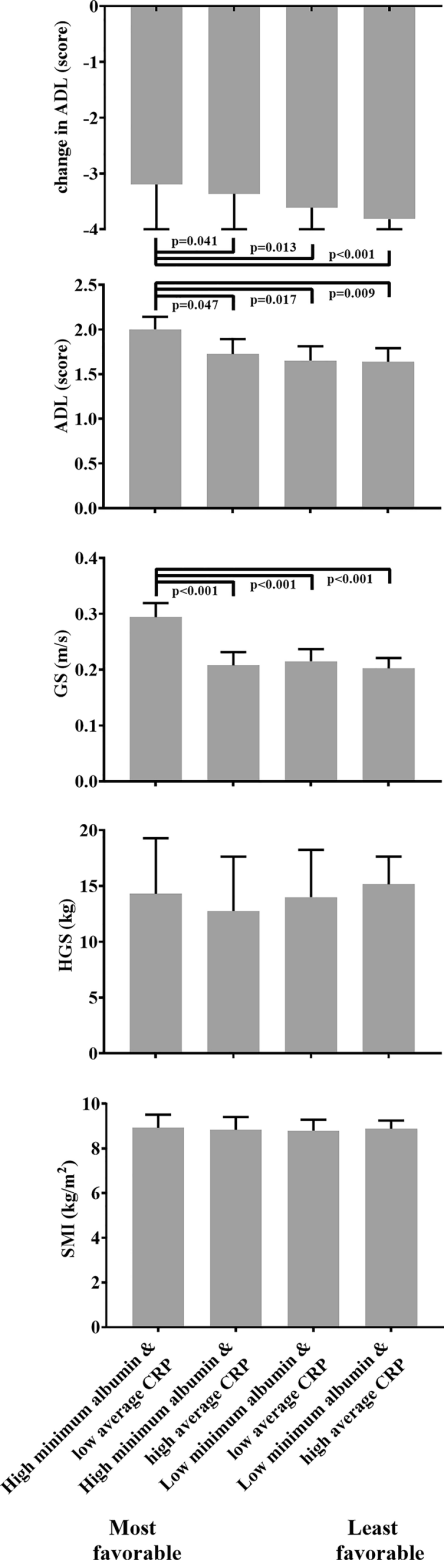
Clusters of minimum albumin and average CRP during acute hospitalization associated with change in ADL, ADL, GS, HGS and SMI at geriatric rehabilitation admission. *ADL* activities of daily living, *CRP* C-reactive protein, *GS* gait speed, *HGS* handgrip strength, *SMI* skeletal muscle mass index. Bars: unstandardized predicted medians adjusted for age, sex and length of acute hospital stay. Change in ADL additionally adjusted for baseline ADL 2 weeks before acute hospitalization. Error bars: upper interquartile range. Low minimum albumin < 29 g/L, high average CRP ≥ 32.4 mg/L

Figure 3 visualizes clusters of minimum albumin and average CRP. Compared to the most favorable cluster (high minimum albumin, low average CRP), the lower favorable clusters with low minimum albumin or high average CRP showed a larger decline in ADL, a lower ADL and GS. No statistically significant associations were observed for HGS and SMI. The clusters of albumin variation and average CRP are visualized in Online Resource 1. Compared to the most favorable cluster (low albumin variation and low average CRP), the least favorable cluster with high albumin variation and high average CRP showed a larger decline in ADL and a lower GS.

## Discussion

This is the first study to investigate the association of acute inflammation during acute hospitalization with changes in ADL, GS, HGS and SMI in patients admitted to geriatric rehabilitation. Albumin exerts a more robust association with functional and body composition parameters compared to CRP.

Previous findings showed that geriatric inpatients with albumin < 35 g/L at admission were more likely to have a decline in ADL from 2 weeks prior to hospitalization to discharge, and they were more likely to stay ADL dependent after 1 year of follow-up [39]. Albumin < 30 g/L at admission to the hospital was associated with a higher risk of new onset ADL disability at discharge in geriatric hospitalized patients [40]. Change in albumin from admission to discharge was positively associated with change in ADL in geriatric patients, as opposed to change in CRP which was not associated with change in ADL [41]. These results from previous studies further support our finding that albumin concentrations have a stronger association with physical function compared to CRP.

CRP was not associated with most functional and body composition parameters, contradicting previous findings from the EMPOWER study in geriatric inpatients [42] that identified a maximum CRP > 10 mg/L was associated with lower HGS at discharge, but not with lower skeletal muscle mass. However, a second study in geriatric inpatients identified patients with CRP > 10 mg/L at admission and every 7th day thereafter had significantly lower HGS at admission compared to patients with low CRP, but HGS was not significantly different at discharge [43]. CRP and HGS assessed at admission were not associated in 33 geriatric inpatients admitted with acute infection-induced inflammation [44]. These findings suggest that CRP concentrations during acute hospitalization are unlikely to act as a reliable marker for predicting declines in physical function during acute hospitalization.

In previous research, clusters of albumin (cutoff point: 3.5 g/dL) and CRP (cutoff point: 5 mg/dL) in geriatric inpatients were associated with higher risk of in-hospital mortality [45]. In patients with sepsis aged 18 years and older, albumin/CRP ratios at admission and discharge were better predictors of 90-day and 180-day mortality after hospitalization compared to albumin or CRP alone [46, 47]. We explored combining albumin and CRP into clusters, and observed an association with functional and body composition parameters. The benefit of combining albumin and CRP predicting relevant clinical outcome needs further research.

The impact of acute hospitalization on geriatric rehabilitation is a highly understudied field, while geriatric rehabilitation patients are at high risk of poor muscle status [48], which is related to worse recovery [49, 50]. In geriatric patients after acute hospitalization, albumin and CRP have been associated with negative rehabilitation outcome and mortality [51, 52]. Albumin and CRP are nonspecific markers of inflammation, but may also have a direct effect on muscle health: the size of myotubes in vitro may decrease after exposure to CRP with a decreased rate of protein synthesis through a decrease in regulators of mechanistic target of rapamycin complex 1 and an increase in phosphorylated AMP-activated protein kinase [53]. Albumin binds pro-inflammatory cytokines and reactive oxygen species, indirectly moderating the effect of systemic inflammation on deterioration [17]. Albumin further activates the phosphatidyl–inositol 3-kinase/AKT pathway [54], potentially leading to muscle hypertrophy [55]. Optimal treatment for geriatric inpatients with inflammation comprises pharmacological, resistance training and nutritional intervention [56–58], but guidelines for personalised training protocols have still to be established.

Some limitations of this study should be considered. Albumin and CRP are not specific markers of inflammation as they also respond to other biological influences. Ideally inflammation should be assessed using pro-inflammatory cytokines, such as IL-6, however; these measurements are not part of routine patient care and as such other markers of inflammation were not accessible for this study. Patients with a higher number of albumin and CRP measurements were more frail, which could have led to selection bias. Not all variables that might influence the association between CRP, albumin and functional and body composition parameters, such as diagnosis, comorbidities and medication use, were investigated. This needs to be further investigated in future research. We did not have data available on use of specific anti-inflammatory medication. Finally, because of the inclusion of patients at admission to geriatric rehabilitation, we were unable to analyze GS, HGS and SMI before acute hospitalization.

In conclusion, in patients admitted to geriatric rehabilitation, higher inflammation, as measured by lower albumin and higher CRP, during acute hospitalization was related to lower functional and body composition parameters at the start of rehabilitation. Particularly, lower average, higher variation and lower minimum albumin were associated with a larger decline in physical function during acute hospitalization.

## Supplementary Information

Below is the link to the electronic supplementary material.

Supplementary file1 (TIF 8977 KB)

Supplementary file2 (DOCX 18 KB)

Supplementary file3 (DOCX 31 KB)

## Data Availability

The data sets during and/or analyzed during the current study available from the corresponding author on reasonable request.
