# Supplementary figures and images for: Albumin and C-reactive protein relate to functional and body composition parameters in patients admitted to geriatric rehabilitation after acute hospitalization: findings from the RESORT cohort
Source: Eur Geriatr Med. 2022 Mar 2;13(3):623–32. doi: 10.1007/s41999-022-00625-5 (PMC9151554; doi:10.1007/s41999-022-00625-5)

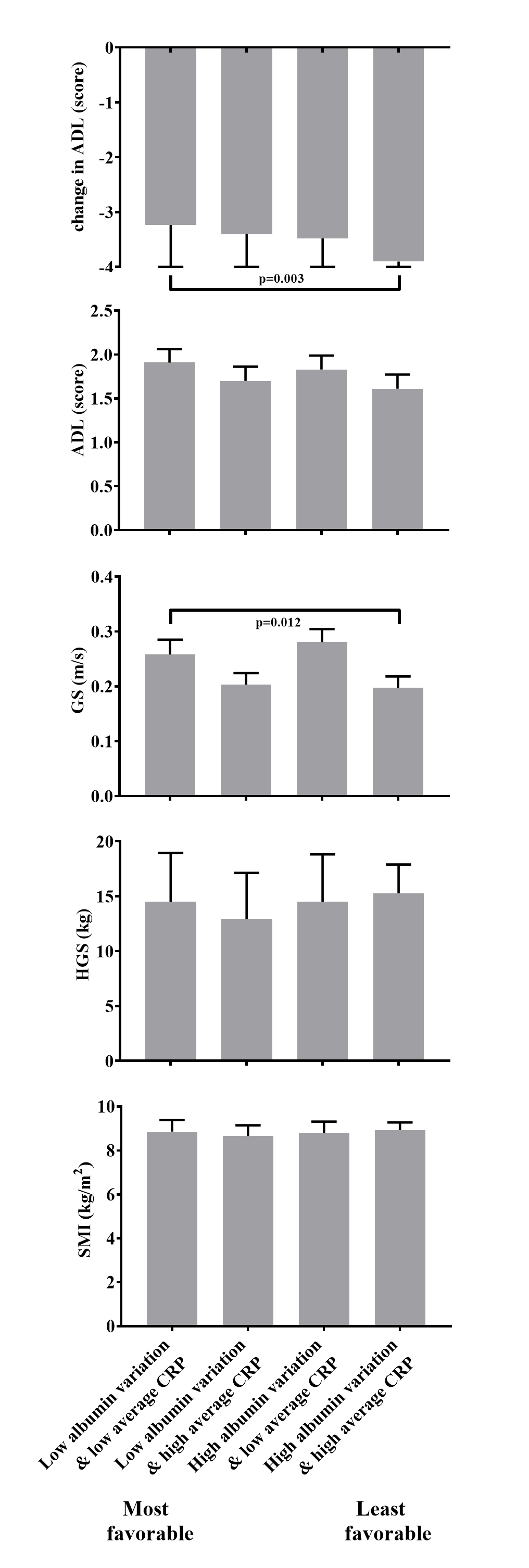

Supplement: Supplementary file 1 — Supplementary file1 (TIF 8977 KB) [file 41999_2022_625_MOESM1_ESM.tif]
