# Supplementary material for: Albumin and C-reactive protein relate to functional and body composition parameters in patients admitted to geriatric rehabilitation after acute hospitalization: findings from the RESORT cohort
Source: Eur Geriatr Med. 2022 Mar 2;13(3):623–32. doi: 10.1007/s41999-022-00625-5 (PMC9151554; doi:10.1007/s41999-022-00625-5)
Supplement: Supplementary file 2 — Supplementary file2 (DOCX 18 KB) [file 41999_2022_625_MOESM2_ESM.docx]

**Online Resource 1 Supplementary Figure.** Clusters of albumin variation and average CRP during acute hospitalization associated with change in ADL, ADL, GS, HGS and SMI at geriatric rehabilitation admission.

ADL: Activities of daily living. CRP: C-reactive protein. GS: Gait speed. HGS: Handgrip strength. SMI: Skeletal muscle mass index.

Bars: Unstandardized predicted medians, adjusted for age, sex and length of acute hospital stay. Change in ADL additionally adjusted for baseline ADL two weeks before acute hospitalization. Error bars: Upper interquartile range. Low albumin variation <2 g/L, high average CRP ≥32.4 mg/L.
