# Supplementary material for: Albumin and C-reactive protein relate to functional and body composition parameters in patients admitted to geriatric rehabilitation after acute hospitalization: findings from the RESORT cohort
Source: Eur Geriatr Med. 2022 Mar 2;13(3):623–32. doi: 10.1007/s41999-022-00625-5 (PMC9151554; doi:10.1007/s41999-022-00625-5)
Supplement: Supplementary file 3 — Supplementary file3 (DOCX 31 KB) [file 41999_2022_625_MOESM3_ESM.docx]

| **Online Resource 2 Supplementary Table.** Patient characteristics stratified by number of albumin and CRP measurements during acute hospitalization | | | | | | | | | | | | | | |
| --- | --- | --- | --- | --- | --- | --- | --- | --- | --- | --- | --- | --- | --- | --- |
|  | | 0 albumin  (n = 30) | | 1 albumin  (n =348) | | ≥ 2 albumin  (n = 1391) | |  | 0 CRP  (n = 383) | | 1 CRP  (n = 437) | | ≥ 2 CRP  (n = 949) | |
| Age, years | | 82.6 (9.3) | | 83.5 (8.2) | | 82.4 (8.0) | |  | 83.0 (7.9) | | 83.2 (8.0) | | 82.1 (8.2) | |
| Sex, female | | 17, 56.7 | | 213, 61.2 | | **754, 54.2** ^b^ | |  | 246, 64.2 | | 256, 58.6 | | **482, 50.8** ^a,b^ | |
| BMI, kg/m^2^ | | 27.5 (6.0) | | 27.6 (6.8) | | **26.5 (6.1)** ^b^ | |  | 26.9 (5.5) | | 26.7 (6.4) | | 26.7 (6.5) | |
| Living independently | | 29, 96.7 | | 324, 93.1 | | 1300, 93.5 | |  | 350, 91.4 | | 409, 93.6 | | 894, 94.2 | |
| CCI score | | 2.1 (2.3) | | 2.4 (2.1) | | **2.9 (2.4)** ^b^ | |  | 2.5 (2.3) | | 2.5 (2.1) | | **3.1 (2.4)** ^a,b^ | |
| Cognitive impairment | | 20, 66.7 | | 237, 68.1 | | 903, 64.9 | |  | 244, 63.7 | | 298, 68.2 | | 618, 65.1 | |
| MST score | | 1 [0–2] | | 0 [0–2] | | **1 [0–2]** ^b^ | |  | 0 [0–2] | | 1 [0–2] | | **1 [0–2]** ^a^ | |
| GLIM malnourished | | 13, 56.5 | | 152, 50.8 | | **640, 57.9** ^b^ | |  | 146, 46.2 | | **213, 58.4** ^a^ | | **446, 59.8** ^a^ | |
| CFS score | | 6 [5–7] | | 6 [5–7] | | **6 [5–7]** ^b^ | |  | 6 [5–7] | | 6 [5–7] | | **6 [5–7]** ^a,b^ | |
| Use of walking aid | | 22, 73.3 | | 254, 73.6 | | 985, 71.3 | |  | 274, 72.3 | | 313, 72.6 | | 674, 71.2 | |
| Fall in previous 12 months | | 20, 66.7 | | 238, 69.4 | | 886, 64.9 | |  | 278, 73.5 | | **278, 64.8** ^a^ | | **588, 63.2** ^a^ | |
| Medication, number | | 9.4 (4.8) | | 8.5 (4.3) | | **9.8 (4.3)** ^b^ | |  | 9.2 (4.1) | | 8.8 (4.5) | | **10.1 (4.2)** ^a^**^,^**^b^ | |
| LOS acute hospitalization, days | | 3 [2–5] | | 4 [2–6] | | **9 [6–15]** ^a^**^,^**^b^ | |  | 6 [3–9] | | **5 [3–8]** ^a^ | | **10 [6–17]** ^a b^ | |
| LOS geriatric rehabilitation, days | | 18 [9–28] | | 19 [13–29] | | **20 [14–33]** ^b^ | |  | 19 [13–31] | | 19 [13–28] | | **21 [14–34]** ^b^ | |
| Albumin, g/L | Average | NA | | 34 [31–36] | | **30 [26–33]** ^b^ | |  | 32 [29–35] | | **33 [30–35]** ^a^ | | **29 [25–33]** ^a^**^,^**^b^ | |
|  | Variation | NA | | NA | | 2 [1–4] | |  | 2 [1–3] | | 2 [1–3] | | **2 [2–4]** ^a,b^ | |
|  | Minimum | NA | | 34 [31–36] | | **27 [23–31]** ^b^ | |  | 30 [27–34] | | **32 [28–34]** ^a^ | | **26 [22–30]** ^a^**^,^**^b^ | |
| CRP, mg/L | Average | 21.8  [10.7–79.0] | | 17.2  [3.2–49.7] | | **37.3**  **[11.0–85.9]** ^b^ | |  | NA | | 10.0  [2.6–37.6] | | **47.4**  **[17.3–98.2]** ^b^ | |
|  | Variation | 12.9  [1.8–93.9] | | 9.4  [3.3–43.0] | | **26.1**  **[6.9–60.5]** ^b^ | |  | NA | | NA | | 24.0  [6.2–59.1] | |
|  | Maximum | 24.9  [10.7–80.1] | | 19.5  [3.5–64.3] | | **62.7**  **[16.1–167.3]** ^a^**^,^**^b^ | |  | NA | | 10.0  [2.6–37.6] | | **87.0**  **[31.9–192.8]** ^b^ | |
| ADL two weeks before acute hospitalization | | 5 [4–6] | | 6 [4–6] | | 6 [4–6] | |  | 6 [5–6] | | **6 [4–6]** ^a^ | | **6 [4–6]** ^a^ | |
| ADL at geriatric rehabilitation admission | | 2 [1–4] | | 2 [1–3] | | **2 [1–2]** ^b^ | |  | 2 [1–3] | | 2 [1–3] | | **1 [1–2]** ^a^**^,^**^b^ | |
|  | | Male | Female | Male | Female | Male | Female |  | Male | Female | Male | Female | Male | Female |
| GS, m/s | | 0.30 (0.36) | 0.14 (0.20) | 0.28 (0.31) | 0.21 (0.26) | 0.26 (0.31) | 0.21 (0.27) |  | 0.30 (0.32) | 0.20 (0.27) | 0.30 (0.33) | 0.22 (0.27) | 0.25 (0.30) | 0.21 (0.26) |
| HGS, kg | | 22.4 (7.4) | 14.3 (3.5) | 19.4 (9.3) | 13.0 (6.9) | 19.0 (9.8) | 11.9  (6.9) |  | 21.3  (9.0) | 13.3 (6.5) | 19.5 (9.9) | 12.7 (6.8) | **18.4 (9.7)** ^a^ | **11.3 (7.0)** ^a^**^,^**^b^ |
| SMI, kg/m^2^ | | 9.57 (1.11) | 8.72 (1.23) | 9.81 (1.49) | 8.66 (1.36) | **9.36 (1.43)** ^b^ | 8.44 (1.32) |  | 9.50 (1.28) | 8.58 (1.15) | 9.62 (1.39) | 8.42 (1.33) | 9.37 (1.51) | 8.51 (1.41) |
| All variables are presented as mean (SD), median [IQR] or n, %. ADL: Activities of daily living. BMI: Body mass index. CCI: Charlson comorbidity index. CFS: Clinical frailty scale. CRP: C-reactive protein. GLIM: Global leadership initiative on malnutrition. GS: Gait speed. HGS: Handgrip strength. IQR: Interquartile range. LOS: Length of stay. MST: Malnutrition screening tool. SD: Standard deviation. SMI: Skeletal muscle mass index.  ^a^ Significantly different vs 0 measurements.  ^b^ Significantly different vs 1 measurement.  **Bold** indicates statistical significant difference (p≤0.05). | | | | | | | | | | | | | | |
